# Admission prognostic nutritional index predicts prolonged hospitalization in severe odontogenic deep neck infections

**DOI:** 10.1007/s10266-025-01142-0

**Published:** 2025-07-04

**Authors:** Eiji Iwata, Kyoichi Obata, Shogo Kikuta, Naoki Kaneko, Kotaro Sato, Norio Kitagawa, Yohei Takeshita, Katsuhisa Matsuo, Junsei Sameshima, Akira Tachibana, Shintaro Kawano, Jingo Kusukawa, Masaya Akashi, Joe Iwanaga, Soichiro Ibaragi

**Affiliations:** 1 Department of Oral and Maxillofacial Surgery, Graduate School of Medicine, Dentistry and Pharmaceutical Sciences, Okayama University, 2-5-1 Shikata-cho, Kitaku, Okayama, 700-8525 Japan; 2 Department of Oral and Maxillofacial Surgery, Kakogawa Central City Hospital, Kakogawa, Japan; 3 Department of Oral and Maxillofacial Surgery, Kobe University Graduate School of Medicine, Kobe, Japan; 4 Dental and Oral Medical Center, Kurume University School of Medicine, Kurume, Japan; 5 Section of Oral and Maxillofacial Oncology, Division of Maxillofacial Diagnostic and Surgical Sciences, Faculty of Dental Science, Kyushu University, Fukuoka, Japan; 6 Department of Oral and Maxillofacial Surgery, Nagoya University Graduate School of Medicine, Nagoya, Japan; 7 Department of Oral and Maxillofacial Anatomy, Graduate School of Medical and Dental Sciences, Institute of Science Tokyo, Tokyo, Japan; 8 Department of Oral and Maxillofacial Radiology, Dentistry and Pharmaceutical Sciences, Okayama University Graduate School of Medicine, Okayama, Japan; 9 Department of Neurology, Tulane Center for Clinical Neurosciences, Tulane University School of Medicine, New Orleans, LA USA

**Keywords:** Odontogenic deep neck infections, Nutrition status, Prognostic nutritional index, Prolonged hospitalization, Multiple spaces with abscess

## Abstract

**Objectives:**

Severe odontogenic deep neck infections (DNIs) can be life threatening. This study investigated the nutritional status of affected patients and evaluated the usefulness of the Prognostic Nutritional Index (PNI) at admission in helping maxillofacial surgeons identify, at presentation, those likely to require extended hospitalization.

**Methods:**

A total of 112 patients treated for odontogenic deep neck abscesses and necrotizing soft tissue infections at five hospitals in Japan. Patients were included. Patients were categorized by length of hospitalization duration and factors associated with prolonged hospitalization were analyzed using propensity score matching to minimize bias. Spearman’s rank correlation analysis was also performed to assess the relationship between PNI and hospitalization duration.

**Results:**

Fifty patients (44.6%) required hospitalization for more than 14 days. Multivariate analysis identified PNI ≤ 41.2 (odds ratio [OR] = 2.79) and the presence of abscesses in multiple deep neck spaces (OR = 2.76) as significant predictors of prolonged hospitalization. Propensity score analysis confirmed the significant association between PNI and length of hospitalization duration (*P* = 0.048). In addition, Spearman’s rank correlation coefficient was *r* = − 0.471 (*P* < 0.001), indicating a moderate negative correlation.

**Conclusion:**

The admission PNI may serve as a useful adjunctive indicator for predicting prolonged hospitalization in patients with severe odontogenic DNIs, as it reflects both nutritional status and systemic inflammation.

## Introduction

Deep neck infections (DNIs) are serious bacterial infections that affects the deep cervical spaces and fascial planes of the neck [1]. Although their incidence has declined with the development of antibiotics, DNIs can still lead to severe complications, including upper airway obstruction, mediastinitis, septic shock, and vascular thrombosis, resulting in significant morbidity and mortality [2]. Among DNIs, deep neck abscesses and necrotizing soft tissue infections (NSTIs) are particularly severe and potentially fatal, and are commonly classified as “severe DNIs” [3, 4]. Deep neck abscesses are localized collections of pus within the deep neck spaces that can present with symptoms ranging from mild pain and fever to life-threatening conditions such as upper airway obstruction and septic shock [5]. NSTIs, on the other hand, are rapidly progressive bacterial infections that spread along multiple fascial planes [6]. These infections may cause vascular compromise, thrombosis, rupture, and necrosis of adipose, muscular, subcutaneous, and cutaneous tissues [6]. Odontogenic infections are recognized as the most common source of DNIs (38.8–49%) [7]. We previously reported the utility of the Laboratory Risk Indicator for Necrotizing Fasciitis (LRINEC) score in diagnosing odontogenic NSTI [8]. Although prompt diagnosis is essential to saving a patient’s life, maxillofacial surgeons also recognize the importance of predicting clinical outcome. Although several studies have investigated prognostic risk factors in DNIs of tonsillar origin [9–11], to our knowledge, no studies have examined the prognosis of patients with severe odontogenic DNIs specifically. Patients with severe odontogenic DNIs often experience malnutrition due to trismus and dysphagia, which impair oral intake [12]. Therefore, this study focused on evaluating their nutritional status.

The Prognostic Nutritional Index (PNI) is an integrated biomarker reflecting both nutritional and inflammatory status, calculated using serum albumin levels and lymphocyte counts [13]. Initially introduced by Buzby et al. in 1980 to assess the nutritional and immunological status of patients undergoing gastrointestinal surgery [14], a simplified version relying solely on serum albumin levels and peripheral lymphocyte counts was later proposed by Onodera et al. in 1984 and has since been widely adopted [15]. Numerous studies have demonstrated that PNI is a valuable prognostic marker in various malignancies [16, 17], and it is now believed to more accurately reflect systemic inflammation and nutritional deficits [18, 19]. Based on these insights, we hypothesized that the admission PNI could serve as an adjunctive predictor of prolonged hospitalization in patients with severe odontogenic DNIs, given their high levels of inflammation and malnutrition. Early prognostic assessment could support more personalized and timely therapeutic strategies.

## Methods

### Patients

This study included 112 patients (aged ≥ 18 years, both sexes) with abscess formation in the deep neck spaces, confirmed by contrast-enhanced computed tomography (CT), who underwent incisional drainage, including debridement of necrotic tissues, at five institutions in Japan between January 2012 and March 2023. At the time of admission all patients underwent blood tests and contrast-enhanced CT. Hospitalization criteria included clinical signs such as skin erythema, dysphagia, difficulty eating, and elevated inflammatory markers in blood tests. Patients were excluded if they did not undergo incisional drainage, or contrast-enhanced CT, or if they did not consent to participate in this study. In all patients, incisional drainage and debridement of necrotic tissues were performed, and the drained pus or necrotic tissue was submitted for bacterial culture on the day of admission. Tracheotomy was performed when severe laryngeal edema was observed or when deemed necessary by the attending physician. All patients received intravenous antibiotics starting from the day of admission. The specific antibiotics used were determined by the attending physician. Broad-spectrum antibiotics were typically selected for empiric therapy and were adjusted based on the antimicrobial susceptibility test results. Intravenous antibiotics were continued until the white blood cell count normalized, *C*-reactive protein (CRP) level significantly decreased, and purulent drainage was no longer observed. Wounds were irrigated daily until drainage ceased. Patients were discharged after the completion of intravenous antibiotic therapy and improvement in their general condition. Based on the length of hospitalization duration, patients were divided into two groups: short term and long term. The long-term group included patients who required hospitalization for more than 14 days, following criteria from previous studies [20, 21].

### Data collection

The following variables were retrospectively reviewed and evaluated from medical records and CT images: patient’s age, sex, presence of diabetes mellitus (DM), smoking history, CRP levels in blood tests, PNI, abscess location in each deep neck space, presence of gas production, presence of tracheostomy, type of anesthesia used during drainage (general or local), presence of causative treatment during hospitalization, duration of intravenous antibiotic therapy, type of initial antibiotics administered, and hospitalization duration. The PNI was calculated using the following formula proposed by Buzby et al.: 158–(16.6 × serum albumin [g/dl])–(0.78 × triceps skinfold [mm])–(0.22 × serum transferrin [mg/dl])–(5.8 × cutaneous delayed hypersensitivity reactivity [0,1,2]). On the other hand, the PNI by Onodera et al. was calculated using the following formula: 10 × serum albumin (g/dl) + 0.005 × peripheral lymphocyte count (per mm^3^) [13, 15–19]. We used the latter in this study. Abscess location and extent were independently analyzed using contrast-enhanced CT images by five observers, one from each institution. CT scans were performed using nine different CT systems, including the 64-slice CT system (Aquilion 64; Canon Medical Systems Corp, Tochigi, Japan) and the 128-slice CT system (SOMATOM Definition Flash; Siemens, Munich, Germany). The data were acquired under standard head and neck CT protocols (120 kV, 1–5 mm slice) with automatic exposure control. Four different contrast agents were used, including Iomeron 300 (Eisai, Tokyo, Japan) and Iopamirdol 370 (Hikari Pharmaceutical, Tokyo, Japan).

### Ethical approval

The study protocol adhered to the ethical principles of the Declaration of Helsinki and the Ethical Guidelines for Medical and Health Research involving Human Subjects issued by the Ministry of Health, Labor, and Welfare of Japan. Ethical approval was obtained from the Institutional Review Board (IRB) (Kurume University Hospital Ethics Review Committee. No. 23122). As this was a retrospective study, all identifiable patient information was anonymized. In accordance with IRB instructions, the research plan was published on the website of each participating hospital, along with an opt-out option for patients.

### Statistical analysis

All statistical analyses were performed using SPSS (version 26.0; SPSS, Chicago, IL, USA) and Ekuseru–Toukei 2016 software (Social Survey Research Information Co. Ltd., Tokyo, Japan). Receiver-operating characteristic (ROC) curves were used to determine cutoff values for age, CRP and PNI, and the area under the ROC curve (AUC) was calculated to assess discrimination accuracy. Associations between each variable and prolonged hospitalization were analyzed using the Mann–Whitney *U* test for ordinal variables and either Fisher’s exact test or the chi-square test for categorical variables. Statistical significance was set at *P* < 0.05. Variables significantly associated with prolonged hospitalization were entered into a multiple logistic regression model using the forced-entry method. Before introduction, multicollinearity was assessed, and variables that did not significantly fit the model were excluded. A goodness-of-fit analysis was performed following the regression. The presence of multicollinearity among variables was evaluated using the Variance Inflation Factor (VIF). Odds ratios (OR) and 95% confidence intervals (CIs) were calculated. To minimize selection bias inherent in retrospective data analysis, propensity score matching was performed between the low and high PNI groups based on the determined cutoff value. Matched cases (*n* = 58) were then analyzed to assess the association between PNI and the hospitalization duration. In addition, Spearman’s rank correlation analysis was also conducted to examine the relationship between PNI and the length of hospitalization duration.

## Results

Of the 112 patients, 50 (44.6%) were included in the long-term group, defined as requiring hospitalization for more than 14 days. This criterion was based on not only on previous studies [20, 21] but also on the median of hospitalization duration observed in the present study. Table 1 shows the patient characteristics and results of the univariate analysis. In the univariate analysis, the prevalence of DM (*P* = 0.024) and elevated CRP levels (*P* = 0.023) were significantly higher in the long-term group than in the short-term group. The PNI was significantly lower in the long-term group (*P* < 0.001). The number of deep neck spaces with abscesses was also significantly greater in the long-term group, both as a continuous variable (*P* = 0.004) and categorical variable (*P* < 0.001). Among the affected spaces, the parotid space, temporal or infratemporal fossa, submasseteric space, and pterygomandibular space were significantly more frequently involved in the long-term group. Conversely, abscesses in the sublingual space were significantly more frequent in the short-term group. Retropharyngeal space abscesses (*n* = 2) were observed only in the long-term group, and both affected patients underwent required tracheostomy. There were no significant differences between groups in anesthesia type during drainage or in the administration of causative treatments during hospitalization. As expected, both the duration of intravenous antibiotic therapy and the hospitalization duration were longer in the long-term group. Although not statistically significant, there was variability across and within institutions in the selection of antibiotics. The most commonly used initial antibiotics in both groups were combination of sulbactam/ampicillin (SBT/ABPC) and clindamycin (CLDM), followed by SBT/ABPC and ceftriaxone (CTRX). These three regimens accounted for over 70% of patients in both groups, with SBT/ABPC used in approximately half. These antibiotics were selected as empirical treatments and adjusted based on antimicrobial susceptibility test results.

**Table 1:** Comparison between long-term and short-term groups

Variable		Long-term group (*n* = 50)	Short-term group (*n* = 62)	*P* value
Age (years)	Median (range)	66.0 (21–93)	61.0 (19–95)	0.197
Sex	Male	27 (54.0%)	32 (51.6%)	0.850
DM	Yes	11 (22.0%)	4 (6.5%)	0.024*
Smoking	Yes	17 (34.0%)	23 (37.1%)	0.843
CRP (mg/dL)	Median (range)	16.3 (1.7–43.4)	12.0 (1.5–30.4)	0.023*
PNI	Median (range)	38.7 (23.5–52.1)	43.4 (25.4–57.6)	< 0.001*
Number of deep neck space with abscess	Median (range)	2.0 (1–6)	1.0 (1–6)	< 0.001*
	Multiple (≥ 2)	37 (74.0%)	29 (46.8%)	0.004*
Submandibular space abscess	Yes	30 (60.0%)	38 (61.3%)	1.000
Sublingual space abscess	Yes	4 (8.0%)	17 (27.4%)	0.014*
Submental space abscess	Yes	13 (26.0%)	18 (29.0%)	0.833
Parotid space abscess	Yes	9 (18.0%)	3 (4.8%)	0.033*
Temporal fossa/infratemporal fossa abscess	Yes	14 (28.0%)	2 (3.2%)	< 0.001*
Sub-masseteric space abscess	Yes	24 (48.0%)	14 (22.6%)	0.006*
Pterygomandibular space abscess	Yes	20 (40.0%)	11 (17.7%)	0.011*
Parapharyngeal space abscess	Yes	7 (14.0%)	4 (6.5%)	0.214
Retropharyngeal space abscess	Yes	2 (4.0%)	0 (0.0%)	0.197
Gas production	Yes	9 (18.0%)	5 (8.1%)	0.153
Tracheotomy	Yes	2 (4.0%)	0 (0.0%)	0.197
Anesthesia when drainage	General	23 (46.0%)	22 (35.5%)	0.333
Causative treatment during hospitalization	Yes	21 (42.0%)	26 (41.9%)	1.000
Duration of intravenous antibiotics (days)	Median (range)	12.0 (4–105)	7.0 (2–12)	< 0.001*
SBT/ABPC + CLDM		13 (26.0%)	18 (29.0%)	0.794
SBT/ABPC		12 (24.0%)	13 (21.0%)	
CTRX		11 (22.0%)	14 (22.6%)	
FMOX		7 (14.0%)	8 (12.9%)	
CZOP		1 (2.0%)	3 (4.8%)	
CLDM		2 (4.0%)	2 (3.2%)	
TAZ/PIPC + CLDM		2 (4.0%)	0 (0.0%)	
MEPM + CLDM		1 (2.0%)	0 (0.0%)	
MEPM		1 (2.0%)	1 (1.6%)	
CEZ		0 (0.0%)	1 (1.6%)	
CMZ		0 (0.0%)	1 (1.6%)	
PZFX		0 (0.0%)	1 (1.6%)	
Hospitalization duration (days)	Median (range)	19.0 (15–105)	10.0 (4–14)	< 0.001*

Bold was defined as “*P* < 0.05”

DM: Diabetes mellitus. CRP: C-reactive protein. PNI: Prognostic nutritional index. SBT/ABPC: sulbactam/ampicillin. CLDM: clindamycin. CTRX: ceftriaxone. FMOX: flomoxef. CZOP: cefozopran. TAZ/PIPC: tazobactam/piperacillin. MEPM: meropenem. CEZ: cefazoline. CMZ: cefmetazole. PZFX: pazufloxacin

*P < 0.05

**Fig. 1:**
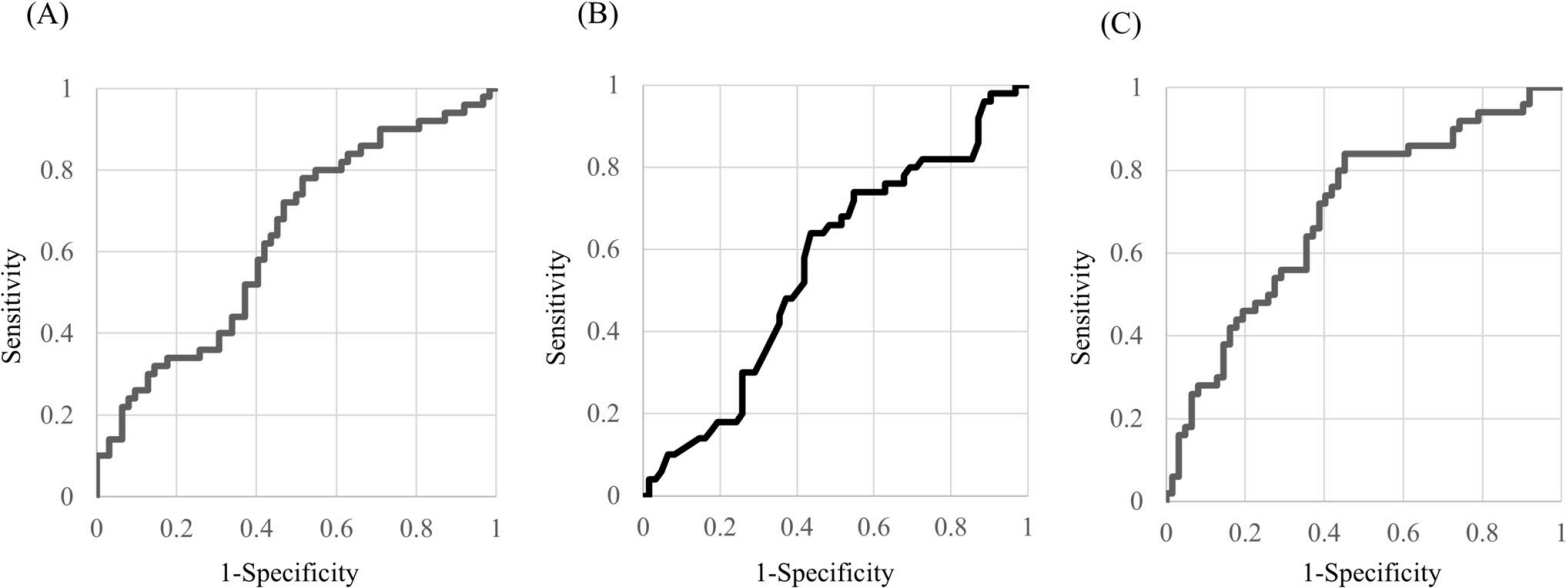
The ROC curves for predicting prolonged hospitalization. **a** The ROC curve for accuracy of age in predicting prolonged hospitalization. The AUC for our model was 0.561 (95% confidence interval 0.453–0.669). **b** The ROC curve for accuracy of CRP in predicting prolonged hospitalization. The AUC for our model was 0.632 (95% confidence interval 0.528–0.736). **c** The ROC curve for accuracy of PNI in predicting prolonged hospitalization. The AUC for our model was 0.696 (95% confidence interval 0.598–0.795)

ROC curve analysis was used to determine cutoff values for three variables. Age ≥ 64 years had a sensitivity of 64.0%, specificity of 56.5%, and AUC of 0.561 (Fig. 1a). CRP ≥ 12.6 mg/dL had a sensitivity of 72.0%, specificity of 53.3%, and AUC of 0.63 (Fig. 1b). PNI ≤ 41.2 had a sensitivity of 72.0%, specificity of 61.3%, and AUC of 0.79 (Fig. 1c). Before performing multiple logistic regression, multicollinearity was assessed using the VIF, which was below 1.5 for all variables, indicating no multicollinearity. Therefore, no variables were excluded. Multivariate analysis revealed that PNI ≤ 41.2 (OR = 2.79) and multiple deep neck spaces involvement (OR = 2.76) were significantly associated with prolonged hospitalization (Table 2). The Hosmer–Lemeshow goodness-of-fit test yielded a *P* value of 0.818, indicating adequate model fit.

**Table 2:** Results of the multivariate logistic regression analysis of the risk factors for prolonged hospitalization

Variable	*P* value	Odds ratio	95% CI	
			Lower	Upper
Age ≥ 64 years (vs. < 64 years)	0.244	1.777	0.676	4.672
Male (vs. Female)	0.734	1.178	0.457	3.038
DM (vs. Absence)	0.377	1.957	0.442	8.668
Smoking (vs. No smoking)	0.714	0.824	0.293	2.316
CRP ≥ 12.6 mg/dL (vs. < 12.6 mg/dL)	0.126	2.026	0.820	5.002
PNI ≤ 41.2 (vs. > 41.2)	0.029*	2.787	1.109	7.006
Multiple deep neck spaces with abscess (vs. Single space)	0.036*	2.756	1.069	7.107
Gas production (vs. Absence)	0.573	1.504	0.365	6.200
Tracheotomy (vs. Absence)	0.999	177,222,036.4	0.000	–
General anesthesia (vs. Local anesthesia)	0.623	1.280	0.478	3.428
Causative treatment during hospitalization (vs. Absence)	0.436	0.680	0.258	1.796

Bold was defined as *P < 0.05

DM: Diabetes mellitus. CRP: C-reactive protein. PNI: Prognostic nutritional index

**Fig. 2:**
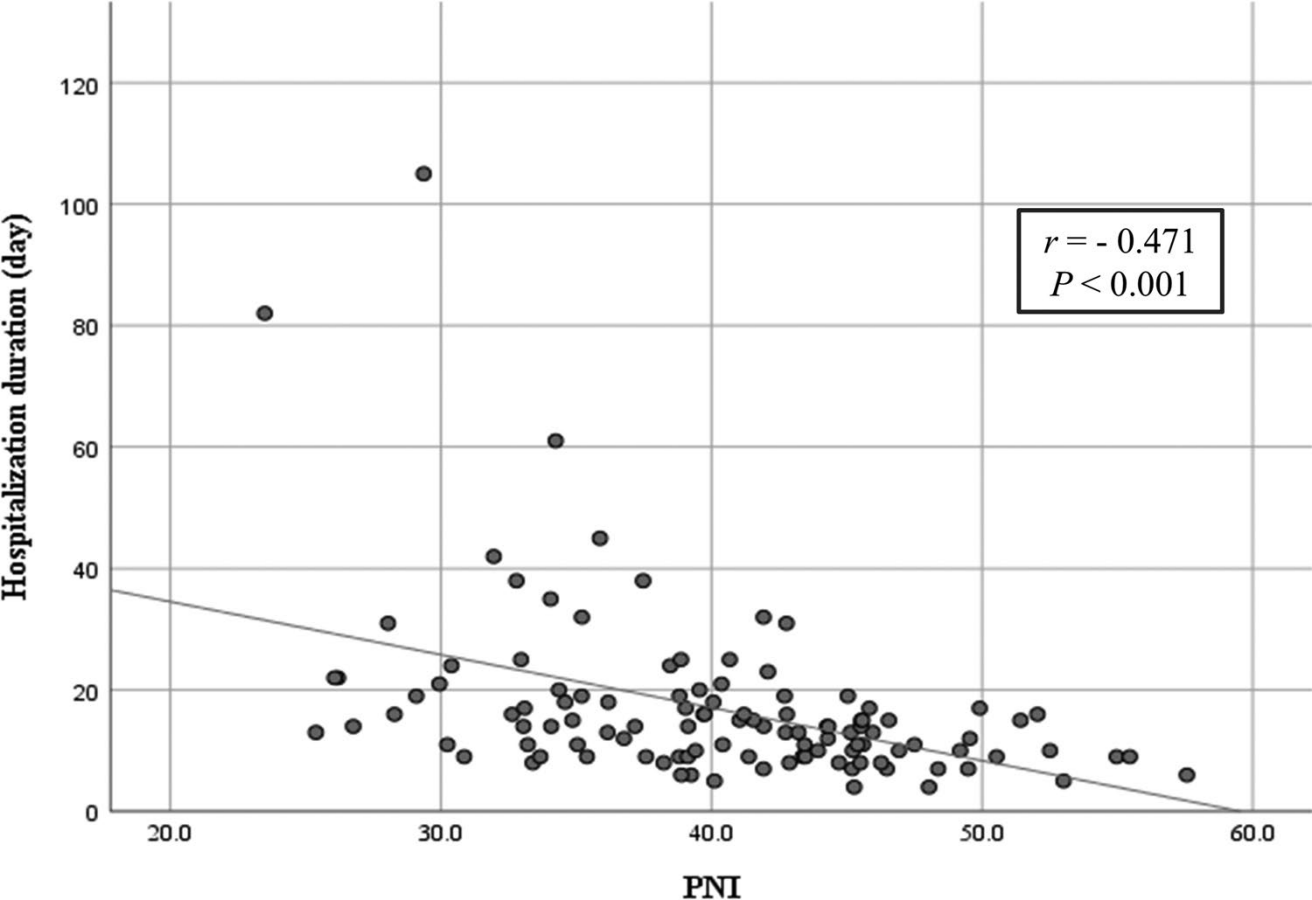
Correlation between PNI and the length of hospitalization duration. **a** significant negative correlation was observed between PNI and hospitalization duration, as calculated using Spearman’s rank correlation coefficient (*r* = − 0.471, *P* < 0.001)

Table 3 compares patient characteristics between the low PNI (≤ 41.2) and high PNI (> 41.2) groups. Low PNI was significantly associated with higher CRP levels (*P* = 0.002) and greater number of deep neck spaces with abscesses (*P* < 0.001). To reduce selection bias, propensity score matching was performed using nine variables (age, sex, DM, CRP, number of deep neck space with abscess, gas production, anesthesia when drainage, and causative treatment during hospitalization). After matching, the two groups were balanced across these variables, and PNI remained significantly associated with the length of hospitalization duration (*P* = 0.048) (Table 4). Figure 2 shows a scatter plot depicting the relationship between PNI and the length of hospitalization duration. Spearman’s rank correlation coefficient was *r* = − 0.471, indicating a moderate negative correlation, with a statistically significant *P* value < 0.001.

**Table 3:** Background factors of patients with low and high PNI

Variable		Low PNI group (*n* = 60)	High PNI group (*n* = 52)	*P* value
Age	Median (range)	70.5 (21–95)	54.5 (19–93)	< 0.001*
Sex	Male	29 (48.3%)	30 (57.7)	0.348
DM	Yes	10 (16.7%)	5 (9.6%)	0.405
Smoking	Yes	18 (30.0%)	22 (42.3%)	0.236
CRP (mg/dL)	Median (range)	17.2 (1.7–43.4)	12.0 (1.5–30.9)	0.002*
Number of deep neck space with abscess	Median (range)	2.0 (1–6)	2.0 (1–6)	< 0.001*
Gas production	Yes	11 (18.3%)	3 (5.8%)	0.051
Tracheotomy	Yes	2 (3.3%)	0 (0.0%)	0.498
Anesthesia when drainage	General	35 (58.3%)	32 (61.5%)	0.847
Causative treatment during hospitalization	Yes	27 (45.0%)	20 (38.5%)	0.566

Bold was defined as *P < 0.05

DM: Diabetes mellitus. CRP: C-reactive protein. PNI: Prognostic nutritional index

**Table 4:** Background factors of patients with low and high PNI after propensity score matching

Variable		Low PNI group (*n* = 29)	High PNI group (*n* = 29)	*P* value
Age	Median (range)	63.0 (21–95)	64.0 (19–93)	0.640
Sex	Male	16 (55.2%)	17 (58.6%)	1.000
DM	Yes	4 (13.8%)	4 (13.8%)	1.000
Smoking	Yes	9 (31.0%)	15 (51.7%)	0.182
CRP (mg/dL)	Median (range)	15.6 (1.7–30.4)	12.5 (2.2–30.9)	0.566
Number of deep neck space with abscess	Median (range)	2.0 (1–6)	2.0 (1–6)	0.896
Gas production	Yes	2 (6.90%)	3 (10.3%)	1.000
Tracheotomy	Yes	0 (0.0%)	0 (0.0%)	1.000
Anesthesia when drainage	General	20 (69.0%)	20 (69.0%)	1.000
Causative treatment during hospitalization	Yes	9 (31.0%)	15 (51.7%)	0.182
Hospitalization duration (days)	Median (range)	16.0 (5–82)	11.0 (4–32)	0.048*

Bold was defined as *P < 0.05

DM: Diabetes mellitus. CRP: C-reactive protein. PNI: Prognostic nutritional index

Table 5 summarizes the bacterial culture results. The detection rate was 92.0% (46/50) in the long-term group and 80.6% (50/62) in the short-term group. In both groups, the most frequently identified bacteria were *Streptococcus* species*,* followed by *Prevotella* species.

**Table 5:** Distribution of microorganisms

Long-term group (46/50)	No. (%)	Short-term group (50/62)	No. (%)
[Facultative anaerobic bacteria]	38 (100.0)	[Facultative anaerobic bacteria]	34 (100.0)
*Streptococcus* spp.	33 (86.8)	*Streptococcus* spp.	28 (82.4)
*S. anginosus*	11	*S. anginosus*	10
*S. constellatus*	9	*S. constellatus*	8
*S. intermedius*	4	*S. intermedius*	2
*S. oralis*	3	*S. epidermidis*	1
*S. mitis*	2	Unidentified species	7
S. cristatus	1	*Staphylococcus* spp.	2 (5.9)
Unidentified species	3	*Actinonomyces* spp.	1 (2.9)
*Staphylococcus* spp.	3 (7.9)	*Eikenella corrodens*	1 (2.9)
*Actinonomyces* spp.	1 (2.6)	*Enterobacter cloacae*	1 (2.9)
*Corynebacterium striatum*	1 (2.6)	*Propinonibacterium* spp.	1 (2.9)
[Obligate anaerobic bacteria]	44 (100.0)	[Obligate anaerobic bacteria]	48 (100.0)
*Prevotella* spp.	23 (52.3)	*Prevotella* spp.	21 (43.8)
*P. buccae*	8	*P. intermedia*	11
*P. intermedia*	7	*P. buccae*	3
*P. corporis*	2	*P. nigrescens*	2
*P. melaninogenica*	2	*P. oralis*	1
*P. nigrescens*	1	*P. denticola*	1
*P. oralis*	1	*P. loescheii*	1
Unidentified species	2	Unidentified species	2
*Fusobacterium* spp.	5 (11.4)	*Parvimonas micra*	9 (18.8)
*Parvimonas micra*	5 (11.4)	*Peptostreptococcus* spp.	8 (16.7)
*Bacteroides* spp.	2 (4.5)	*Fusobacterium* spp.	8 (16.7)
*Porphyromonas* spp.	2 (4.5)	*Veillonella* spp.	1 (2.1)
*Peptostreptococcus* spp.	2 (4.5)	*Micromonas* spp.	1 (2.1)
*Veillonella* spp.	1 (2.3)		
*Finegoldia magna*	1 (2.3)		
*Solobacterium moorei*	1 (2.3)		
*Atopobium parvulum*	1 (2.3)		
*Filifactor alocis*	1 (2.3)		

The detection rate per group was 46/50 for the long-term group and 50/62 for short-term group

## Discussion

The main purpose of this study was to evaluate whether the admission PNI could serve as an adjunctive indicator for predicting prolonged hospitalization in patients with severe odontogenic DNIs. Among the 112 patients included, 50 (44.6%) patients required long-term hospitalization > 14 days. Multivariate analysis revealed that PNI ≤ 41.2 (OR = 2.79) and the presence of abscesses in multiple deep neck spaces (OR = 2.76) were significantly associated with long-term hospitalization. Additionally, propensity score analysis demonstrated a significant association between PNI and the length of hospitalization duration (*P* = 0.048). Spearman’s rank correlation coefficient was* r* = − 0.471, indicating a moderate negative correlation, with statistical significance (P < 0.001).

We focused on PNI as an objective indicator of nutritional status. Our findings indicate that lower admission PNI is significantly associated with prolonged hospitalization in patients with severe odontogenic DNIs. In general, nutritional status is closely linked to patient prognosis [22]. Although PNI was originally introduced to assess cancer patients’ prognoses [15–19], its applicability has since expanded to conditions such as heart failure [23] and novel COVID-19 [24]. A recent study have also demonstrated its utility in sepsis prognosis [25]. The PNI incorporates serum albumin and peripheral lymphocyte count, with low values reflecting hypoalbuminemia and lymphocytopenia, indicators of malnutrition and impaired immune function [26]. Therefore, our results support the clinical relevance of admission PNI as an adjunctive marker for anticipating prolonged hospitalization, especially given the nutritional vulnerability often observed in this patient population.

We also analyzed the extent and location of deep neck abscesses. Univariate analysis showed that patients in the long-term group had significantly more abscess-involved spaces than those in the short-term group (median: two vs. one). Multivariate analysis confirmed that involvement of multiple deep neck spaces was an independent predictor of prolonged hospitalization. This finding likely reflects the increased difficulty in managing extensive abscesses, which typically require longer drainage periods. Flynn et al. suggested the severity index of odontogenic DNIs [27, 28]. This index classified DNIs into three risk categories based on the risk of the airway compromise or proximity to vital structures [27, 28]. The low-risk category includes vestibular, subperiosteal, infraorbital, and buccal spaces [27, 28]. The medium-risk category includes sublingual, submandibular, submental, pterygomandibular, submasseteric, and temporal spaces [27, 28]. The high-risk category includes parapharyngeal, retropharyngeal, pre-tracheal, danger space, mediastinum, and intracranial spaces [27, 28]. In this study, the involvement of the parotid, temporal, submasseteric, and pterygomandibular spaces (all medium-risk) was significantly more frequent in the long-term group, though the sublingual space was more frequently involved in the short-term group. Although the sublingual space was more frequently involved in the short-term group. These findings suggest that infections in deeper or less accessible neck spaces may require more complex surgical management and lead to delayed resolution. Among high-risk sites, only the retropharyngeal space showed a notable difference, with both affected patients in the long-term group requiring tracheotomy. This aligns with our previous work, which recommended tracheostomy in patients with retropharyngeal abscesses due to the risk of airway obstruction [4, 29].

In terms of microbiology, *Streptococcus* species were the most frequently detected organisms, followed by *Prevotella*, consistent with previous literature [30, 31]. However, we observed notable variability in antibiotic selection both within and between institutions. Although regional differences in oral microflora necessitate some variation in antibiotic regimens [32], our findings highlight the need for standardized treatment protocols for odontogenic DNIs. Although not a formal guideline, the 2016 recommendations by the Japanese Association for Infectious Diseases and the Japanese Society of Chemotherapy (JAID/JSC 2016) proposed sulbactam/ampicillin (SBT/ABPC) as a first-line agent for treating odontogenic infections due to its efficacy against *Prevotella*, a *β*-lactamase-producing organism [33, 34]. In our study, the most commonly used regimen was a combination of SBT/ABPC and clindamycin (CLDM), followed by SBT/ABPC and ceftriaxone (CTRX). These three regimens accounted for more than 70% of cases in both groups, and SBT/ABPC alone was used in about half. Although the JAID/JSC 2016 guideline did not explicitly recommend combining SBT/ABPC with CLDM, the latter was likely chosen for its excellent tissue penetration, particularly into abscesses [35]. Our findings support the use of SBT/ABPC (with the addition of CLDM) as a reasonable standardized approach to managing severe odontogenic DNIs, especially given the frequent detection of *Prevotella* species.

To our knowledge, this is the first multicenter study investigating the clinical outcomes of patients with severe odontogenic DNIs. We found that lower admission PNI is significantly associated with longer hospitalization. However, this study had several limitations. First, although propensity score matching was employed to reduce bias, the retrospective design inherently limits the ability to control all confounding factors. Second, treatment protocols—including antibiotic selection and nutritional support—were not standardized across institutions, which may have affected outcomes. Recognizing these limitations, we plan to conduct a prospective study with standardized treatment protocols, including antibiotic regimens and airway management, to further validate our findings and contribute to the development of evidence-based clinical guidelines.

In conclusion, admission PNI, derived from routine blood tests, may serve as an adjunctive indicator for predicting prolonged hospitalization in patients with severe odontogenic DNIs, reflecting both nutritional status and systemic inflammation. However, as PNI is only a supplementary tool, patients should still be evaluated comprehensively based on the clinical findings and managed with individualized treatment strategies.

## Data Availability

The datasets can be obtained from the corresponding author upon reasonable request.
